# Supplementary material for: NMR Analysis Suggests Synergy between the RRM2 and the Carboxy-Terminal Segment of Human La Protein in the Recognition and Interaction with HCV IRES
Source: Int J Mol Sci. 2023 Jan 29;24(3):2572. doi: 10.3390/ijms24032572 (PMC9916714; doi:10.3390/ijms24032572)
Supplement: Supplementary file 1 [file ijms-24-02572-s001.zip › ijms-2134913-supplementary.pdf]

NMR analysis suggests synergy between the RRM2 and the carboxy-terminal segment of human La protein in the recognition and interaction with HCV IRES

Aikaterini Argyriou[1], Georgios A. Machaliotis[1], Garyfallia I. Makrynitsa[1], Eleni Kaliatsi[2], Constantinos Stathopoulos[2], Georgios A. Spyroulias[1,*]

1 Department of Pharmacy, University of Patras, GR-26504, Patras, Greece
2 Department of Biochemistry, School of Medicine, University of Patras, GR-26504, Patras, Greece
* Correspondence: G.A.Spyroulias@upatras.gr

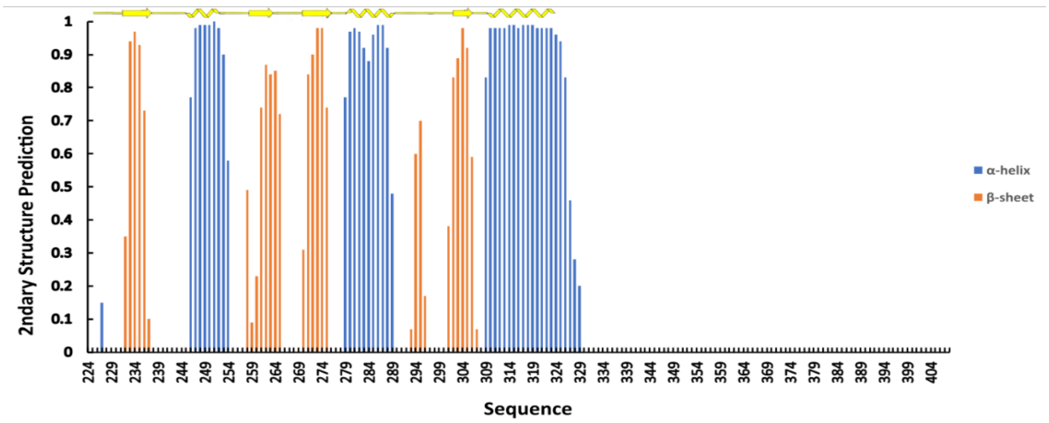

Figure S1: Upper panel: Schematic diagram of secondary structure of human La RRM2 NMR structure (PDB ID: 1OWX) [12]. Lower panel: Secondary structure prediction of NMR analysis of human La RRM2-Cter (La 224-408) derived from TALOS+ server. TALOS+ failed to predict any secondary structure element for the polypeptide spanning residues 330-408, further supporting the disordered nature of the La C-terminal tail.

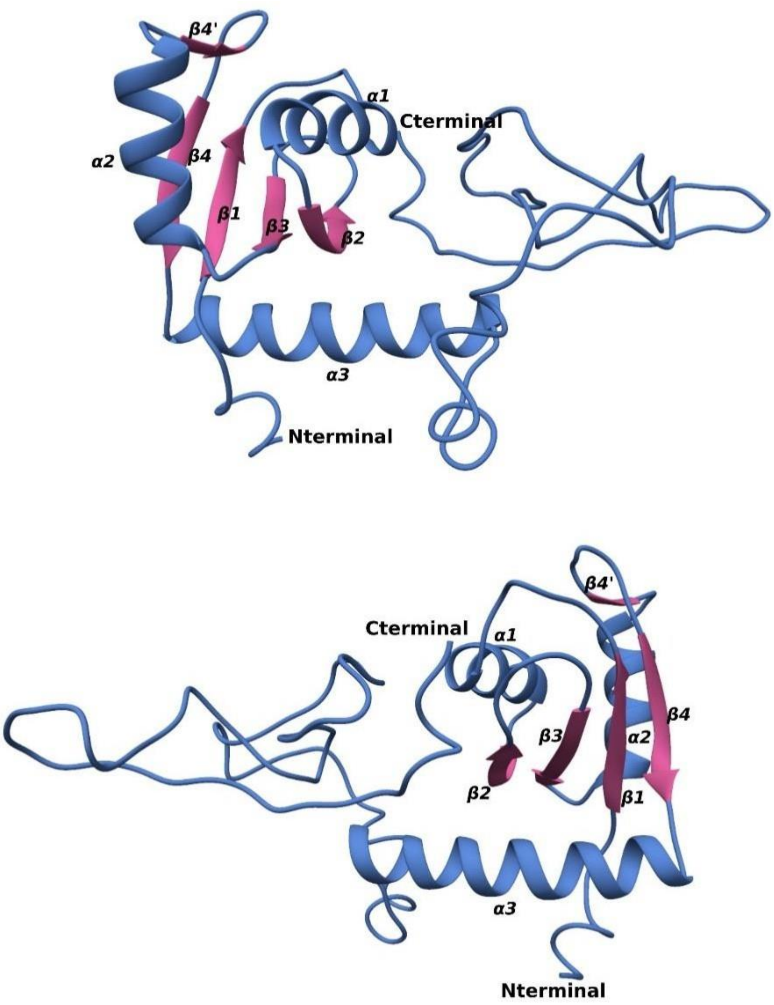

Figure S2: 3D Structure of human La RRM2-Cter (La 224-408) as predicted through server CS Rosetta [26–29] usingthe chemical shifts of the NMR analysis.

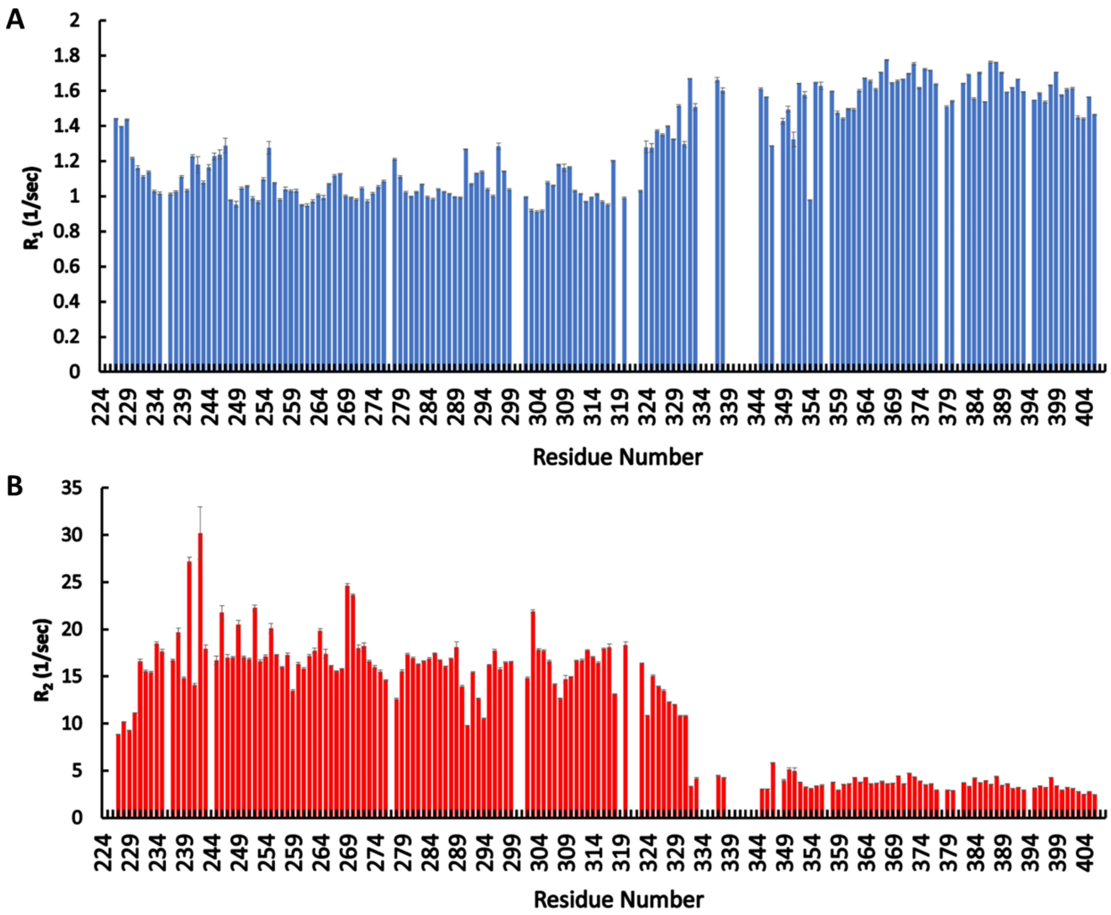

Figure S3: A. R1 values and **B.** R2 values of human La RRM2-Cter (La 224-408).

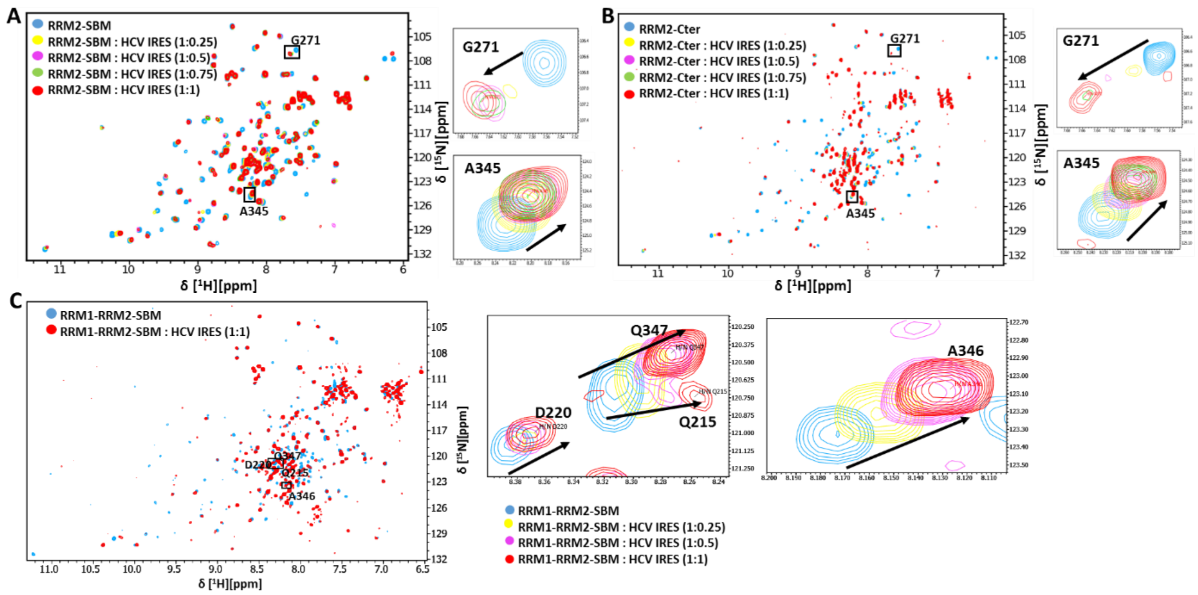

Figure S4: Overlay of the ^{1}H-^{15}N HSQC of the A. RRM2-SBM, B. RRM2-Cter and C. RRM1-RRM2-SBM in apo-state in blue and in protein : HCV-IRES ratio 1:0,25 (in yellow), 1:0,5 (in purple), 1:0,75 (in green) and 1:1 (in red). Magnification of the selected ^{1}H-^{15}N HSQC regions representing examples of the amino acids that found in the fast-exchange regime on the NMR time-scale.

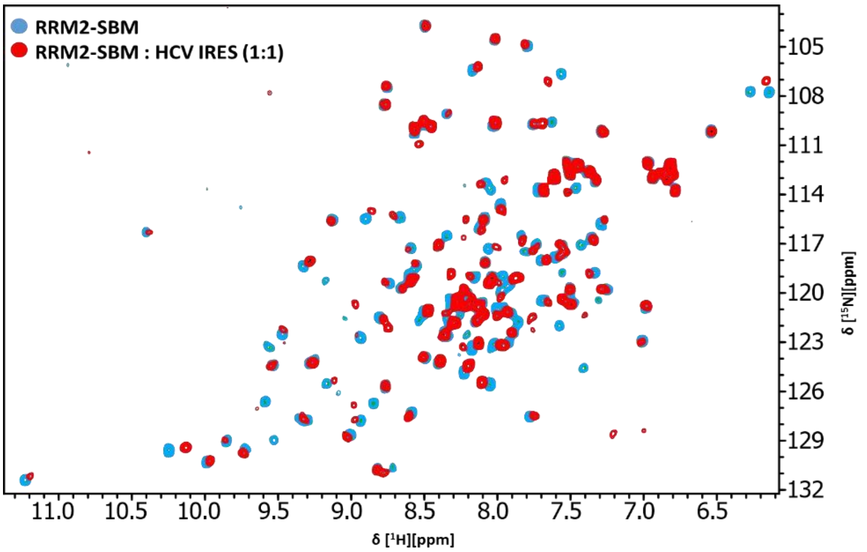

Figure S5: Overlay of ^{1}H-^{15}N HSQC spectra of the hRRM2-SBM domain in the apo state (blue) and bound to HCV-IRES in molar ratio 1.00:1.00 (red).

Supplementary Table S1: Affected amino acids of hRRM2-SBM after the addition of the IRES domain IV of the HCV in molar ratio 1.00:1.00.

$0{,}076 \leq \Delta CS_{N\text{-}H} < 0{,}1$	$0{,}1 \leq \Delta CS_{N\text{-}H}$	*disappeared NH*	*non determined NH*
Trp261, Glu270, Ile318, Asp321, Gln323, Trp329, Lys337, Ala345, Lys352	Cys245, Leu249, Glu258, Gly267, Gly271, Ile272, Ile273, Leu274, Glu277, Leu326	Glu247, His250, Ile262, Asp263, Phe264, Lys332	Met224, Ser225, Leu226, Asp242, Arg246, Val265, Glu300, Ile319, Gln322, Arg334, Phe336, Lys339, Gly340, Lys341, Gly342, Asn343, Lys344, Pro348, Ser350, Gly351

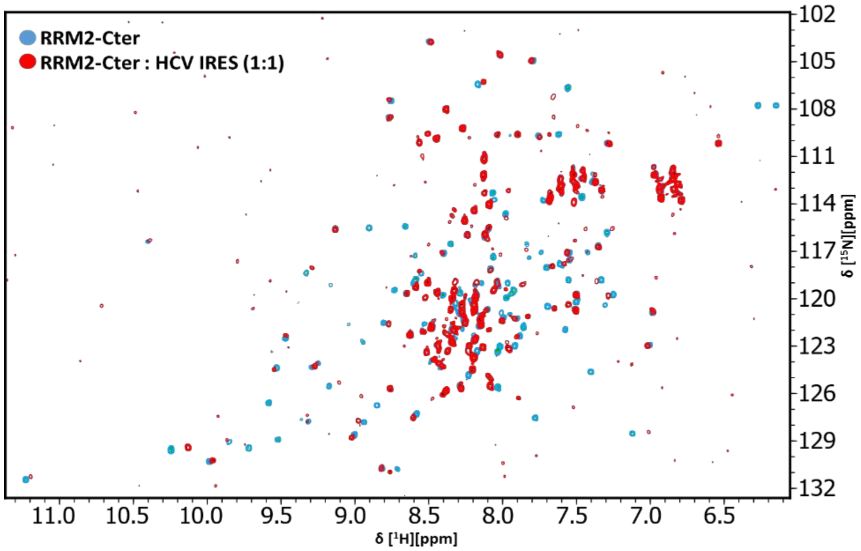

Figure S6: Overlay of ^{1}H-^{15}N HSQC spectra of the hRRM2-Cter domain in the apo state (blue) and bound to HCV-IRES in molar ratio 1.00:1.00 (red).

Supplementary Table S2: Affected amino acids of hRRM2-Cter after the addition of the IRES domain IV of the HCV in molar ratio 1.00:1.00.

0,064≤ΔCS$_{N-H}$<0,1	***0,1≤ΔCS$_{N-H}$***	***disappeared NH***	***non determined NH***
Glu227, Gln243, Thr244, Ser254, Glu270, Ile273, Phe275, Ala279, Ile318, Glu324, Ala345	His250, Glu258, Trp261, Gly267, Gly271, Ile272, Glu277, Gln323	Cys245, Arg246, Glu247, Leu249, Ile262, Asp263, Phe264, Val265, Arg266, Leu274, Leu326, Lys328,	Met224, Ser225, Leu226, Phe236, Asp242, Glu300, Glu309, Ile319, Gln322, Gly333, Arg334, Arg335, Phe336, Lys337, Lys339, Gly340, Lys341, Gly342, Asn343, Lys344, Pro348, Gly349, Gly351, Gly353, Phe357, Lys363, Ala378, Pro381, Pro394, Asp407, Gln408

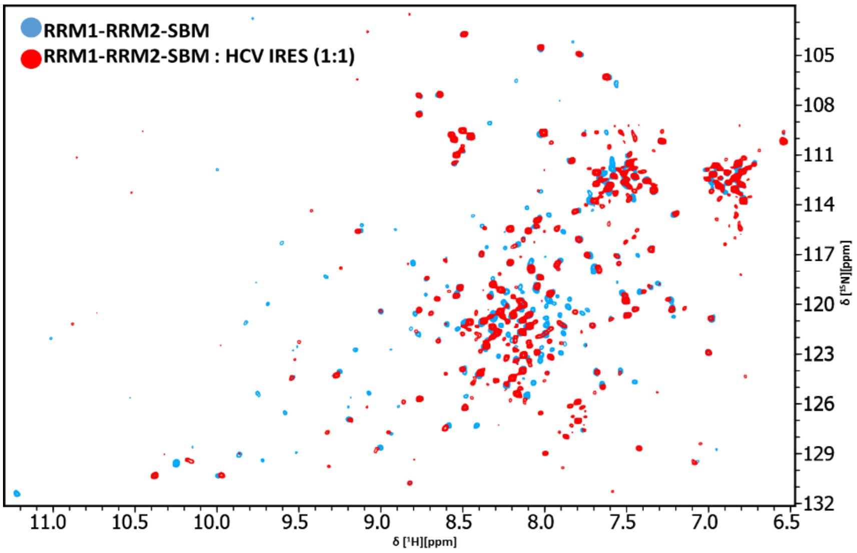

Figure S7: Overlay of ^{1}H-^{15}N HSQC spectra of the hRRM1-RRM2-SBM domain in the apo state (blue) and bound to HCV-IRES in molar ratio 1.00:1.00 (red).

Supplementary Table S3: Affected amino acids of hRRM1-RRM2-SBM after the addition of the IRES domain IV of the HCV in molar ratio 1.00:1.00. The amino acids that belong to RRM1 are in blue, the amino acids that belong to linker between RRM1 and RRM2 are in green and the amino acids that belong RRM2 are in red.

$0{,}04 \le \Delta CS_{N\text{-}H} < 0{,}07$	*$0{,}07 \le \Delta CS_{N\text{-}H}$*	*disappeared NH*	*non determined NH*
Asn110, Val137, Ile140, Phe150, Glu162, Val168, Asn193, Lys204, Leu205, Glu212, Gln215, Glu227, Glu228, Leu233, Asp239, Ser254, Gln295, Thr302, Leu315, Trp329, Ala346, Gln347	Ala203, Glu258, Ile318, Asp321	Val108, Arg111, Val113, Leu138, Met142, Val156, Val157, Phe158, Ile182, Leu183, Lys185, Asp187, Ala190, Val201, Glu218, Lys229, Lys235, Gly238, Leu240, Asp241, Gln243, His250, Phe253, His256, Trp261, Ile262, Asp263, Phe264, Arg266, Gly267, Gly271, Ile272, Ile273, Leu274, Glu277, Ala279, Ala314, Ser325, Leu326, Asn327, Lys328, Lys330, Lys337	Lys105, Asn106, Asp107, Ile115, Lys116, Pro119, Arg143, Arg144, Thr145, Leu146, His147, Lys148, Lys151, Gly152, Phe155, Ile161, Pro171, Lys174, Tyr175, Lys176, Glu177, Leu181, Asp186, Phe189, Lys191, Lys192, Glu194, Glu195, Arg196, Lys197, Gln198, Asn199, Lys200, Glu202, Lys208, Gln209, Glu210, Gln211, Glu219, Ala221, Met223, Lys224, Ser225, Leu226, Gly231, Cys232, Asp242, Cys245, Arg246, Glu247, Lys260, Val265, Lys269, Phe275, Glu300, Glu309, Ile319, Glu320, Gln322, Arg334, Phe336,Lys339, Gly340, Lys341, Gly342, Asn343, Lys344, Pro348, Gly349, Ser350, Gly351, Gly353

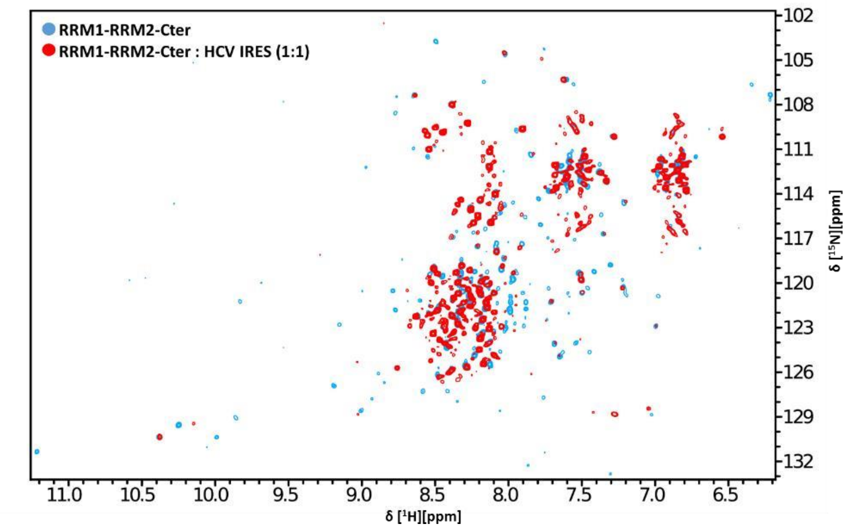

Figure S8: Overlay of the ^{1}H-^{15}N HSQC of the RRM1-RRM2-Cter in apo-state in blue and in protein : HCV-IRES ratio 1:1 in red.

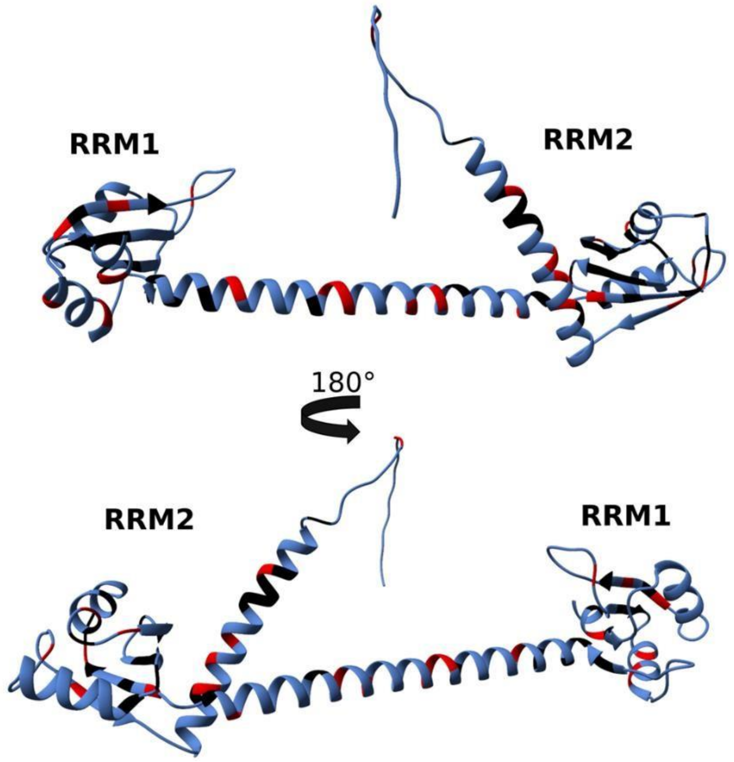

Figure S9: Mapping perturbated (red) and disappeared (black) peaks due to HCV-IRES addition on RRM1-RRM2-SBM (La 105-359) AlphaFold 3D structure of La protein [29,30]

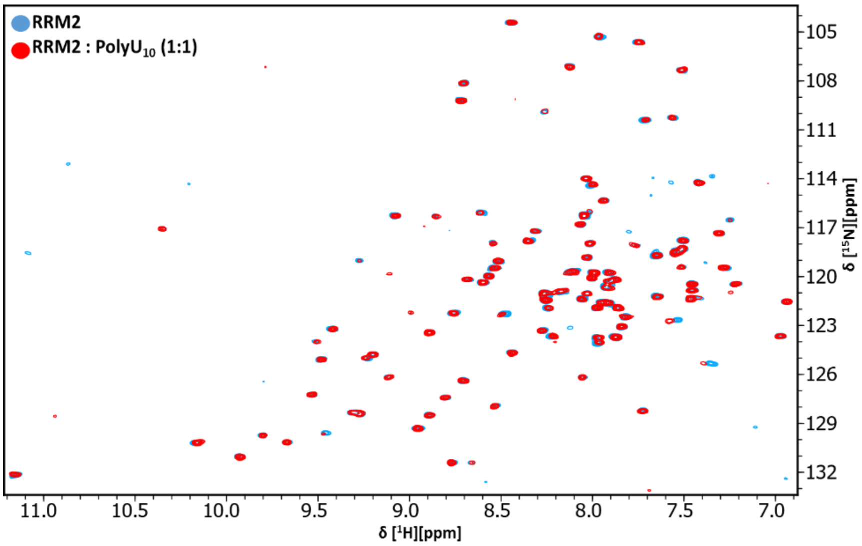

Figure S10: Overlay of ^{1}H-^{15}N HSQC spectra of the hRRM2 domain in the apo state (blue) and bound to HCV-IRES in molar ratio 1.00:1.00 (red).

Supplementary Table S4: Affected amino acids of hRRM2-Cter after the polyU addition in molar ratio 1.00:1.00.

0,01793≤ΔCS$_{N\text{-}H}$<0,1	***0,1≤ΔCS$_{N\text{-}H}$***	***disappeared NH***	***non determined NH***
Glu247, Ile262, Phe264, Val265, Glu277, Ile318			Met224, Ser225, Leu226, Phe236, Asp242, Ser254, Glu300, Ile319, Gln322, Gly333, Arg334

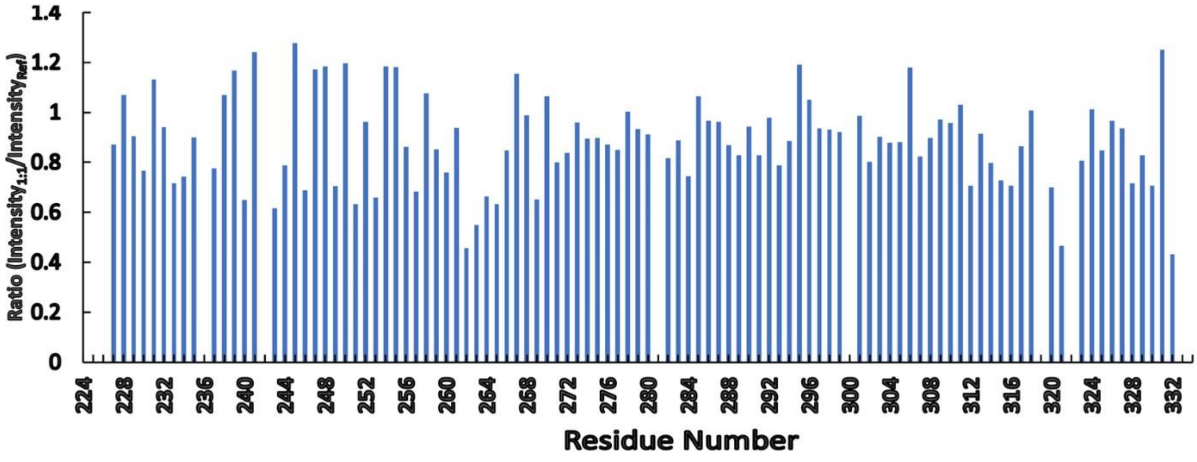

Figure S11: Ratio of peak intensity for each residue of RRM2 domain before and after the addition of the unlabeled ligand PolyU$_{10}$ (Intensity$_{1:1}$/Intensity$_{ref}$). [43]

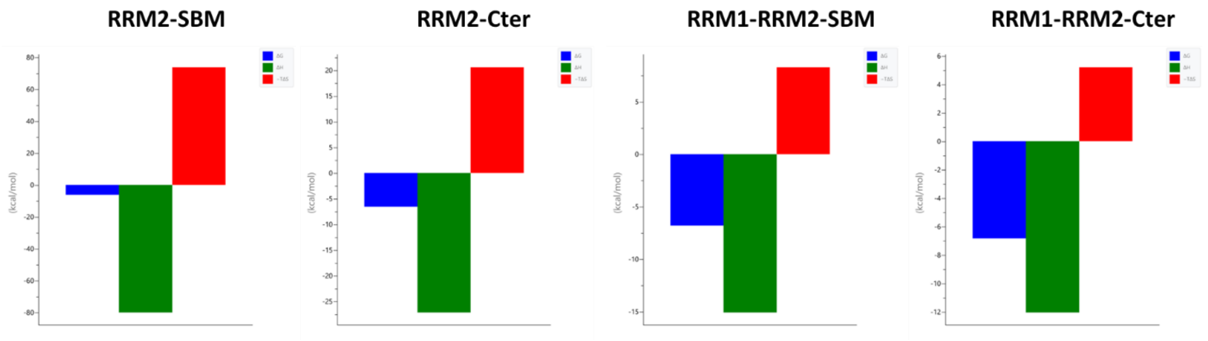

Figure S12: Multiple thermodynamic "signature" plot of the complexes formation (ΔG in blue, ΔH in green and –TΔS in red) determined by ITC titration.

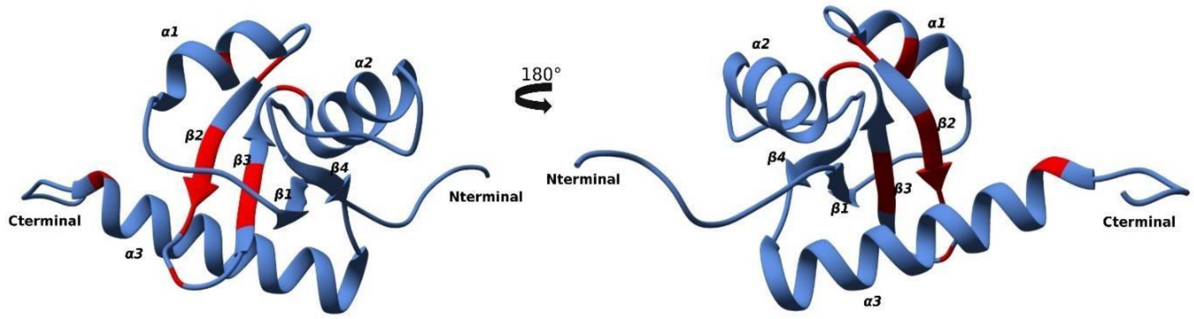

Figure S13: Mapped residues (red) of RRM2 domain that are affected in all titration experiments with HCV-IRES on the 3D NMR structure of La RRM2 domain (1OWX) [12].
